# Genome-wide identification and expression pattern analysis of quinoa BBX family

**DOI:** 10.7717/peerj.14463

**Published:** 2022-12-05

**Authors:** Du Xuefen, Xiaohong Wei, Baoqiang Wang, Zhu Xiaolin, Wang Xian, Luo Jincheng

**Affiliations:** 1 Gansu Agricultural University, Gansu Provincial Key Laboratory of Aridland Crop Science, Gansu, Lanzhou, China; 2 Gansu Agricultural University, College of Life Science and Technology, Gansu, Lanzhou, China; 3 Gansu Agricultural University, College of Agronomy, Gansu, Lanzhou, China

**Keywords:** Quinoa, BBX family, Abiotic stress, Expression pattern analysis

## Abstract

BBX is a transcription factor encoding zinc finger protein that plays a key role in plant growth and development as well as in responding to abiotic stresses. However, in quinoa, which is known as a “super grain” and has extremely high nutritional value, this gene family has not yet been thoroughly studied. In this study, in order to fully understand the family function of the BBX in quinoa, a total of 31 BBX members were identified by bioinformatics methods. These BBX members were mainly acidic proteins, and most of their secondary structures were random coil s, 31 *CqBBX* members were unevenly distributed on 17 chromosomes, and the analysis of replication events found that quinoa BBX genes produced a total of 14 pairs of gene replication. The BBX genes were divided into five subfamilies according to phylogenetics, and its gene structure and conserved motif were basically consistent with the classification of its phylogenetic tree. In addition, a total of 43 light response elements, hormone response elements, tissue-specific expression response elements, and abiotic stress response elements were found in the promoter region, involving stress elements such as drought and low temperature. Finally, the expression patterns of *CqBBX* genes in different tissues and abiotic stresses were studied by combining transcriptome data and qRT-PCR , and all 13 genes responded to drought, salt, and low-temperature stress to varying degrees. This study is the first comprehensive study of the BBX family of quinoa, and its results provide important clues for further analysis of the function of the abiotic stress response.

## Background

Transcription factors (TFs) are important gene-regulating proteins that play a vital role in plant growth and development when subjected to stress (Kim et al., 2021). There are generally two types, one is an activator and the other is an inhibitor, which is combined with a cis-acting element when acting as activation or inhibitor to regulate downstream gene expression (Riechmann et al., 2000). In addition, some factors can act as both activator and repressor when it binds to different positions or binds with different heterodimerization partners (Boyle & Després, 2010). A good example of the potato *PR-10a* gene and *A. thaliana PR-1* and *AtYY1*, which are both dual-function transcription factors with both activation and repression domains (Boyle & Després, 2010; Li et al., 2016). Zinc-finger TF is one of the most important TFs in plants. Zinc-finger proteins contain zinc-finger domains that are stabilized by zinc and other metal ions and can bind to DNA, RNA, or proteins (Khanna et al., 2009). The BBX proteins are one of the important transcriptional regulators encoding zinc finger proteins in plants, which play a great role in responding to light, temperature, plant development, and environmental changes (Robson et al., 2001; Valverde et al., 2004). The BBX TF family is known for having one or two B-box domains at the N-terminus of its proteins, and a few genes have a CCT (Constans, Co-like, and TOC1) protein domain at the C-terminus. Both the conserved B-box domain and the CCT protein domain play important functions (Griffiths et al., 2003). The BBX TF family has been identified in a number of plants, including *Arabidopsis thaliana* (Lyu, Li & Li, 2020), *Gossypium spp* (Feng et al., 2021), *Vitis vinifera* (Wei et al., 2020), *Capsicum annuum* (Kim et al., 2021), *Solanum lycopersicum* (Chu et al., 2016), *Phyllostachys heterocycla* (Ma et al., 2021), and *Oryza sativa* (Huang et al., 2012), but the BBX family has not yet been studied in quinoa (*Chenopodium quinoa* Willd., Cq). An increasing number of evidence that the plant BBX proteins play a key role in different physiological and biochemical processes, such as inducing flowering, photomorphogenesis, shading reaction, carotenoid biosynthesis, and biotic and abiotic stress responses (González-Schain et al., 2012; Lee et al., 2010; Chang, Maloof & Wu, 2011; Lin et al., 2018; Crocco et al., 2011; Xiong et al., 2019; Soitamo et al., 2008; Ding et al., 2018).

Previous studies have found that *CONSTANS (CO)*/*AtBBX1* was the first BBX TF to regulate flowering time by triggering the expression of the *T (FT)* gene at the flowering site in *A. thaliana* (Putterill et al., 1995). The flowering time of CO mutants was significantly delayed under long-day conditions, while the flowering time of co-overexpressed transgenic plants were advanced under both long and short-day conditions (Suárez-López et al., 2001; Samach et al., 2000). At present, proteins encoded by *AtBBX4*, *AtBBX7*, and *AtBBX32* genes have been found to play a major role in regulating the flowering time of plants (Datta et al., 2006; Cheng & Wang, 2005; Tripathi et al., 2017). At least ten BBX family genes have now been identified as early photomorphogenesis regulators in *A. thaliana* (Gangappa & Botto, 2014), some of which were positive and some negative. Among them, the genes that positively regulate photomorphogenesis in plants were *AtBBX4*, *AtBBX21*, *AtBBX22*, and *AtBBX23* (Datta et al., 2006; Xu et al., 2016; Xu et al., 2018; Zhang et al., 2017), and the negative regulators of photomorphogenesis included *AtBBX19*, *AtBBX20*, *AtBBX24*, *AtBBX25*, *AtBBX28* and *AtBBX32* (Lin et al., 2018; Fan et al., 2012; Gangappa, Holm & Botto, 2013; Wang et al., 2015). *MdBBX20* and *MdBBX22* in apples and *PpBBX16* in pears encode proteins with the same domain as *AtBBX1* and could influence light input pathways to actively regulate light-induced anthocyanin accumulation (Bai et al., 2019; Fang et al., 2019; An et al., 2019). In addition, ELONGATED HYPOCOTYL5 (HY5) played a vital role in the development of light-signal transduction (Lee et al., 2007). Multiple BBX TFs were an important part of regulatory networks that contained HY5 and mediate photomorphogenesis. HY5 can activate *At BBX22* and inhibits transcription of *At BBX30* and *At BBX31* because it binds to the G-box cis-element in the promoters, thereby negatively affecting photomorphogenesis (Chang, Maloof & Wu, 2011; Heng et al., 2019). *At BBX21* modulated HY5 activity after transcription and post-transcription to promote photomorphogenesis (Xu et al., 2018; Job et al., 2018). Protein interactions of HY5 with *At BBX23* coordinated the regulation of light-mediated gene expression (Zhang et al., 2017).

The BBX protein is also involved in abiotic stress responses and hormone signal transduction networks. For example, *AtBBX18* and *AtBBX23* were positive regulators of thermal formation, and deletion mutations in *AtBBX18* and *AtBBX23* result in decreased thermal responsive hypocotyl elongation (Ding et al., 2018). In *A. thaliana*, *AtBBX24* (STO) acts as a salt-tolerant protein that enhanced the tolerance of yeast cells to salt (Lippuner, Cyert & Gasser, 1996). In addition, overexpression of *AtBBX24* promoted the growth of *A. thaliana* root systems under high-salt conditions (Nagaoka & Takano, 2003). Previous studies have also shown that *CmBBX22* in members of the Chrysanthemum BBX family was a direct homologous of *AtBBX22*, which was transcribed throughout the plant and induced by water deficiency caused by PEG treatment. *CmBBX22* enhanced cold and drought resistance by delaying leaf senescence in *A. thaliana* (Liu et al., 2019). *AtBBX18* (*AtDBB1a*) positively regulates the gibberellin (GA) signaling pathway and played a major role in plant hormone signaling transduction (Wang et al., 2011), while *AtBBX20* (*AtBZS1*) negatively regulates the canolas lactone signaling network (Fan et al., 2012).

Quinoa is a dicotyledonous annual herb belonging to the genus Chenopodium, originally found in the Andes Mountains of South America (Hong et al., 2017). Quinoa has a very high nutritional value, and its protein content is higher than that of crops such as wheat and corn. In addition, quinoa is also high in vitamins, dietary fiber, sugars, and unsaturated fatty acids (Gordillo-Bastidas et al., 2016; Ng et al., 2007; Nowak, Du & Charrondière, 2016). Currently, although the BBX family has been identified in several species, the BBX TF in quinoa has not been comprehensively studied. In this study, the whole genome of the quinoa BBX was identified and its expression was analyzed, in order to understand the response mechanism of quinoa BBX TF in response to abiotic stress, and lay a foundation for further research on the cloning of the quinoa BBX family and the functional identification of individual genes.

## Materials & Methods

### Plant material and treatment

L-1 (Longli NO.1 from Gansu Academy of Agricultural Sciences, Gansu, China ) was used as the test material, and the disinfection method was as follows: first, sterilize the full seeds with 5% NaClO for 15 min, then rinse them with distilled water for 5–6 times, and put the seeds on a clean surface to dry. The seeds were planted in plastic pots with diameter is 15 cm. After previous irrigation, the plants were cultured at room temperature (day/night temperature (24-37) °C/(16-22) °C, humidity (70% ± 10%)) in the plant Physiology laboratory of Gansu Agricultural University. When the seedlings reached 30 days, the seedlings were thinned, and 10 seedlings with consistent growth were retained in each pot. In addition, to ensure adequate nutrition, 300 mL of 1/2 Hoagland nutrient solution was water. when the seedlings were about 50 days old, the transcripts amounts of *CqBBX* genes under different treatments were detected. Seedlings were treated separately for drought (PEG), salt (NaCl), and low temperature (4 °C), with three sets of replicates for each treatment. The control was the untreated sample at 0 h. For drought and salt stress, plants were watered with PEG6000 (20%, w/v) or 200 mmol ⋅L^−1^ in NaCl solution and grown at normal room temperature. For low-temperature stress, placed the plants in a light incubator at a temperature of 4 °C for incubation. Quinoa seedlings were treated for 0, 3, 6, 9, 12, 24, and 48 h respectively, with 30 plants treated at a time. After treatment, 30 plants were randomly divided into three groups, with 10 plants in each group for seedling sampling. When collecting samples, the collected samples should be rapidly placed in liquid nitrogen, and then after all samples were collected, all samples were stored in a refrigerator at −80 °C for later RNA extraction.

### Identification and sequence analysis of *CqBBX*

The genome sequences and annotation files of quinoa were obtained from the Ensembl Plants (https://plants.ensembl.org/index.html). The *A. thaliana* BBX protein sequences were obtained from the TAIR (https://www.arabidopsis.org/tools/bulk/sequences/index.jsp) websites. The *A. thaliana* BBX protein sequence was used as the reference sequence, based on the conserved domain of B-box (PF06203), and the whole genome protein sequences of quinoa were scanned using the BLAST program (e-value <1e^−5^) of TBtools (version 1.09876) (Chen et al., 2020), 51 genes were obtained. The genes containing known conserved domains were retained and identified on the Pfam (http://pfam.xfam.org/family), SMART (http://smart.embl-heidelberg.de/), and NCBI-CDD (https://www.ncbi.nlm.nih.gov/cdd/) online websites (Lu et al., 2020), and 31 BBX members were finally identified in quinoa.

The FASTA sequence of the BBX proteins was submitted to the ExPASY (https://web.expasy.org/protparam/) website to predict the molecular size, molecular weight, isoelectric point, instability index, fatty acid index, and hydrophobicity index of the BBX family members, and then submitted the Fasta sequence of the BBX proteins to Cell-PLoc 2.0 (http://www.csbio.sjtu.edu.cn/bioinf/Cell-PLoc-2/) website performs subcellular localization analysis of members of the quinoa BBX TF family (Chou & Shen, 2008). In the NPS @ (https://npsa-prabi.ibcp.fr/cgi-bin/npsa_automat.pl?page=npsa_gor4.html) website, the protein secondary structure of members of the BBX TF family of quinoa was predicted, the parameters were default.

### Analysis of chromosome localization and gene replication events

Chromosomal position information of the *CqBBX* genes was obtained based on quinoa genome annotation information (Cq_PI614886_gene_V1_pseudomolecule.gff) and visualized chromosome localization using TBtools software (version 1.09876) (Chen et al., 2020). Based on the Fasta sequence of *CqBBX* genes, the gene replication analysis was performed on NCBI BLAST, and the Ka/Ks value of the duplicated gene pair was calculated to evaluate the evolutionary selection. T = Ks/2X*1000000 (*X* = 1.5/1000000) was used to estimate the time (Millions of years ago, MYA) of replication of each *CqBBX* gene (Lynch & Conery, 2003).

### Multi-sequence alignment and construction of phylogenetic trees

The whole-genome data of quinoa, soybean, and grape were downloaded from the Phytozome and Ensembl Plants respectively to obtain the protein sequences of the BBX gene families of quinoa, soybean, grape, and *A. thaliana*. First, the comparison of amino acid sequences was aligned using the ClustalW program with default parameters (Thompson, Higgins & Gibson, 1994). Then, a maximum-likelihood (ML) phylogenetic tree was constructed using the MEGA 11 program, with bootstrap 1,000 repetitions, and “JTT+G+I” was found to be the best ML model (Tamura, Glen & Kumar, 2021). The evolutionary tree was beautified with Evolview 3.0 (https://www.evolgenius.info/evolview/) (Subramanian et al., 2019).

### Gene structure analysis and prediction of conserved motifs

The BBX gene structure information was extracted from the quinoa genome, and the BBX gene structure information was submitted to the Gene Structure Display Server2.0 (http://gsds.gao-lab.org/index.php) website, and the format was selected GTF/GFF3 to obtain the gene structure map of the quinoa BBX gene family (Hu et al., 2015). Submit the amino acid sequence of quinoa BBX to the MEME (https://meme-suite.org/meme/doc/meme.html) website to predict the conserved motifs of CqBBX proteins, set the parameter to the number of motifs to 10 (Steven, 1996), and the other parameters were set at default.

### Promoter cis-acting element analysis

Sequences 2,000 bp upstream of the transcription initiation site of *CqBBX* gene family members were obtained from the quinoa genome database and submitted to the PlantCARE (http://bioinformatics.psb.ugent.be/webtools/plantcare/html/) website for analysis of the acting elements on the promoter sequences of all BBX gene family members (Lescot et al., 2002), and finally, the cis-acting elements related to light, hormone, tissue-specific expression, and abiotic stress were screened for analysis.

### Protein interaction prediction

The protein sequences of quinoa BBX TF family members were submitted to the STRING (https://string-db.org/) website (Szklarczyk et al., 2015), and the orthologetic genes of BBX in *A. thaliana* were screened out for reference (combined score ≥ 0.6), and the protein interplay networks relationships were further obtained, and the mapping software was used to visualize.

### Expression pattern analysis based on transcriptome data

RNA sequencing data of different tissues and organs of quinoa and seedling tissues of quinoa field under different treatments (drought, low Pi, heat, and salt stress) were downloaded from NCBI sequence reading archives (accession numbers: PRJNA394651 and PRJNA306026). Fastp was used for quality control of downloaded raw reads, and clean reads were filtered to obtain high-quality sequencing data (Chen et al., 2018). HISAT2 software was used for comparison and analysis based on the reference genome (Kim, Ben & Salzberg, 2015). The results were assembled and quantified using StringTie software (Pertea et al., 2015), and FPKM was used to quantify the abundance of all genes in each sample. The data of 31 target genes were screened in Excel and standardized using a Log_2_ FPKM method (Gao et al., 2018). Finally, the normalized data were used to generate heat maps of *CqBBX* gene expression using the heat map software TBtools (version 1.09876).

### Quantitative Reverse Transcription PCR (qRT-PCR) analysis

PrimerPremier 5 online software was used to design primers for fluorescence quantitative PCR of the quinoa BBX TF family (Table S1). The design parameters include amplicon length, 150–200 bp; primer length, 15–25 bp; melting temperature (Tm), 56–70 °C (Li et al., 2019). The *actin* was used as the endogenous control (GeneBank: LOC110715281), and the transcripts amounts of genes from the different treatments were normalized. Total RNA was extracted with RNAex Pro Reagent and cDNA was prepared with the Evo M-MLV kit (Accurate Biotechnology (Hunan) Co., Ltd., Changsha, China). Using reverse transcription of cDNA as a template, qRT-PCR analysis was performed with the ABI-VIIA 7 real-time PCR system (Applied Biosystems, Waltham, MCA, USA) using 2 × quantitect-sybr-green-pcr-mix (QIAGEN China Co., Ltd., Shanghai, China). The total amplification reaction is 20 µL, of which 1 µL of template cDNA, 1 µL of upstream and downstream primers (10 µmol/L), EvaGreen 2 × qPCR MasterMix is 10 µL, and ddH2O is 7 µL. The amplification steps were pre-denaturation at 95 °C for 6 min, denaturation at 95 °C for 10 s, and renaturation at 60 °C for 30 s. A total of 40 cycles were carried out. There were three biological replicates for each qRT-PCR reaction. The comparative Ct (2^−ΔΔCt^) method was used to calculate transcripts amounts. The method of one-way ANOVA was used for comparative analysis between processes with SPSS 22.0 software (IBM, Armonk, NY, USA), and the significant level was *P* < 0.05.

## Results

### Acquisition and identification of sequences of quinoa BBX family members

According to the sequence comparison, a total of 31 BBX members were obtained in quinoa, named *CqBBX01* ∼*CqBBX31* (Table 1). The molecular size and molecular weight of quinoa BBX family proteins ranged from 118 to 472 aa and 13075.74 to 52941.02, respectively, of which the smallest molecular length and molecular weight was *CqBBX30*, with values of 118 aa and 13075.74, and the largest was *CqBBX22*, with values of 472 aa and 52941.02; the isoelectric point was between 4.7 and 8.28, and most of them were weakly acidic proteins, and there are only three proteins with isoelectric points greater than 7, namely *CqBBX03*, *CqBBX26*, and *CqBBX28*; the instability index varies from 32.39 to 61.78, the smallest was *CqBBX13*, and the largest was *CqBBX28*; the fat coefficient was 54.01∼82.11, of which the smallest was *CqBBX11* with a value of 54.01, and the largest was *CqBBX13* with a value of 82.11; the mean hydrophobicity varies from −0.928 to −0.203, indicating that all family members were hydrophilic. Subcellular prediction revealed that all 31 CqBBX proteins were located in the nucleus, further confirming their role in the nucleus.

**Table 1:** Primary structure of the BBX gene family in quinoa.

Gene	Gene locus ID	Size (aa)	Molecular weight (Da)	Isoelectric point	Instability index	Aliphatic index	GRAVY	Subcellular Localization
*CqBBX01*	AUR62023859-RA	243	27010.43	4.92	43.73	71.15	−0.471	Nucleus
*CqBBX02*	AUR62035217-RA	298	32286.17	5.59	41.33	65.34	−0.460	Nucleus
*CqBBX03*	AUR62035221-RA	345	39086.02	8.28	58.26	71.54	−0.757	Nucleus
*CqBBX04*	AUR62041089-RA	362	40966.15	4.70	49.61	69.23	−0.601	Nucleus
*CqBBX05*	AUR62002328-RA	243	27029.56	4.93	41.92	73.17	−0.447	Nucleus
*CqBBX06*	AUR62019649-RA	310	33612.68	5.11	43.21	68.90	−0.372	Nucleus
*CqBBX07*	AUR62030805-RA	467	52801.04	5.07	55.89	56.19	−0.928	Nucleus
*CqBBX08*	AUR62040293-RA	324	35477.35	5.33	35.02	67.78	−0.478	Nucleus
*CqBBX09*	AUR62037849-RA	341	38335.10	4.52	45.94	70.32	−0.495	Nucleus
*CqBBX10*	AUR62009440-RA	361	39966.40	5.22	41.97	55.98	−0.632	Nucleus
*CqBBX11*	AUR62023118-RA	367	40883.47	5.24	41.00	54.01	−0.685	Nucleus
*CqBBX12*	AUR62037140-RA	411	44668.54	5.24	47.70	63.87	−0.523	Nucleus
*CqBBX13*	AUR62034563-RA	190	20842.53	6.57	32.39	82.11	−0.287	Nucleus
*CqBBX14*	AUR62034638-RA	364	40455.66	5.43	48.98	61.87	−0.662	Nucleus
*CqBBX15*	AUR62020307-RA	298	32296.21	5.59	41.10	65.67	−0.464	Nucleus
*CqBBX16*	AUR62002867-RA	403	45998.47	5.82	53.84	64.37	−0.826	Nucleus
*CqBBX17*	AUR62002793-RA	470	52435.78	6.63	51.06	68.06	−0.653	Nucleus
*CqBBX18*	AUR62014436-RA	325	35728.90	6.26	36.66	72.03	−0.453	Nucleus
*CqBBX19*	AUR62039579-RA	310	33522.51	5.09	48.10	69.23	−0.385	Nucleus
*CqBBX20*	AUR62016801-RA	118	13161.87	5.72	40.84	73.56	−0.34	Nucleus
*CqBBX21*	AUR62016828-RA	211	23213.10	5.52	56.28	64.69	−0.512	Nucleus
*CqBBX22*	AUR62014102-RA	472	52941.02	5.89	52.65	69.03	−0.676	Nucleus
*CqBBX23*	AUR62041163-RA	157	16967.93	5.05	39.97	69.62	−0.274	Nucleus
*CqBBX24*	AUR62025058-RA	411	44774.62	5.11	47.19	63.14	−0.545	Nucleus
*CqBBX25*	AUR62039984-RA	362	39860.01	5.62	41.56	67.13	−0.543	Nucleus
*CqBBX26*	AUR62001354-RA	265	28916.79	7.85	55.20	76.49	−0.260	Nucleus
*CqBBX27*	AUR62008578-RA	398	44531.37	5.87	49.72	64.17	−0.675	Nucleus
*CqBBX28*	AUR62025650-RA	260	28289.17	8.12	61.78	78.77	−0.203	Nucleus
*CqBBX29*	AUR62030486-RA	430	48655.58	5.32	57.47	54.42	−0.952	Nucleus
*CqBBX30*	AUR62010415-RA	118	13075.74	5.71	36.20	73.56	−0.310	Nucleus
*CqBBX31*	AUR62010390-RA	177	19636.99	6.07	60.14	67.63	−0.653	Nucleus

From Table 2 it can be seen that the proportion of random coil in the secondary structure of BBX proteins was the majority, while the smallest proportion of random coil was *CqBBX02* and *CqBBX15*, which was 50.16%, and the largest was *CqBBX23*, which was 70.06%. A-helix and extension strand account for a smaller proportion, of which the *α*-helix accounted for the smallest proportion of *CqBBX23*, accounting for only 6.37%, while the least proportion of extended strand was *CqBBX03*, accounting for only 11.30%.

**Table 2:** Secondary structure of the *BBX* gene family in quinoa.

Gene name	Alpha helix(Hh)		Extended strand(Ee)		Random coil(Cc)	
	Number	Proportion/%	Number	Proportion/%	Number	Proportion/%
CqBBX01	49	20.16%	43	17.70%	151	62.14%
CqBBX02	111	36.39%	41	13.44%	153	50.16%
CqBBX03	104	30.14%	39	11.30%	202	58.55%
CqBBX04	105	29.01%	62	17.13%	195	53.87%
CqBBX05	52	21.40%	39	16.05%	152	62.55%
CqBBX06	69	22.26%	44	14.19%	197	63.55%
CqBBX07	154	32.98%	66	14.13%	247	52.89%
CqBBX08	55	16.98%	74	22.84%	195	60.19%
CqBBX09	70	20.53%	71	20.82%	200	58.65%
CqBBX10	97	26.87%	73	20.22%	191	52.91%
CqBBX11	98	26.70%	69	18.80%	200	54.50%
CqBBX12	76	18.49%	79	19.22%	256	62.29%
CqBBX13	26	13.68%	38	20.00%	126	66.32%
CqBBX14	54	14.84%	57	15.66%	253	69.51%
CqBBX15	111	36.39%	41	13.44%	153	50.16%
CqBBX16	118	29.28%	61	15.14%	224	55.58%
CqBBX17	152	32.34%	73	15.53%	245	52.13%
CqBBX18	56	17.23%	52	16.00%	217	66.77%
CqBBX19	58	18.71%	44	14.19%	208	67.10%
CqBBX20	20	16.95%	19	16.10%	79	66.95%
CqBBX21	48	22.75%	42	19.91%	121	57.35%
CqBBX22	147	31.14%	77	16.31%	248	52.54%
CqBBX23	10	6.37%	37	23.57%	110	70.06%
CqBBX24	90	21.90%	69	16.79%	252	61.31%
CqBBX25	44	12.15%	65	17.96%	253	69.89%
CqBBX26	59	22.26%	62	23.40%	144	54.34%
CqBBX27	68	17.09%	57	14.32%	273	68.59%
CqBBX28	64	24.62%	65	25.00%	131	50.38%
CqBBX29	126	29.30%	63	14.65%	241	56.05%
CqBBX30	20	16.95%	19	16.10%	79	66.95%
CqBBX31	36	20.34%	42	23.73%	99	55.93%

### Chromosomal localization and repeat events of the Quinoa BBX gene family

**Figure 1:**
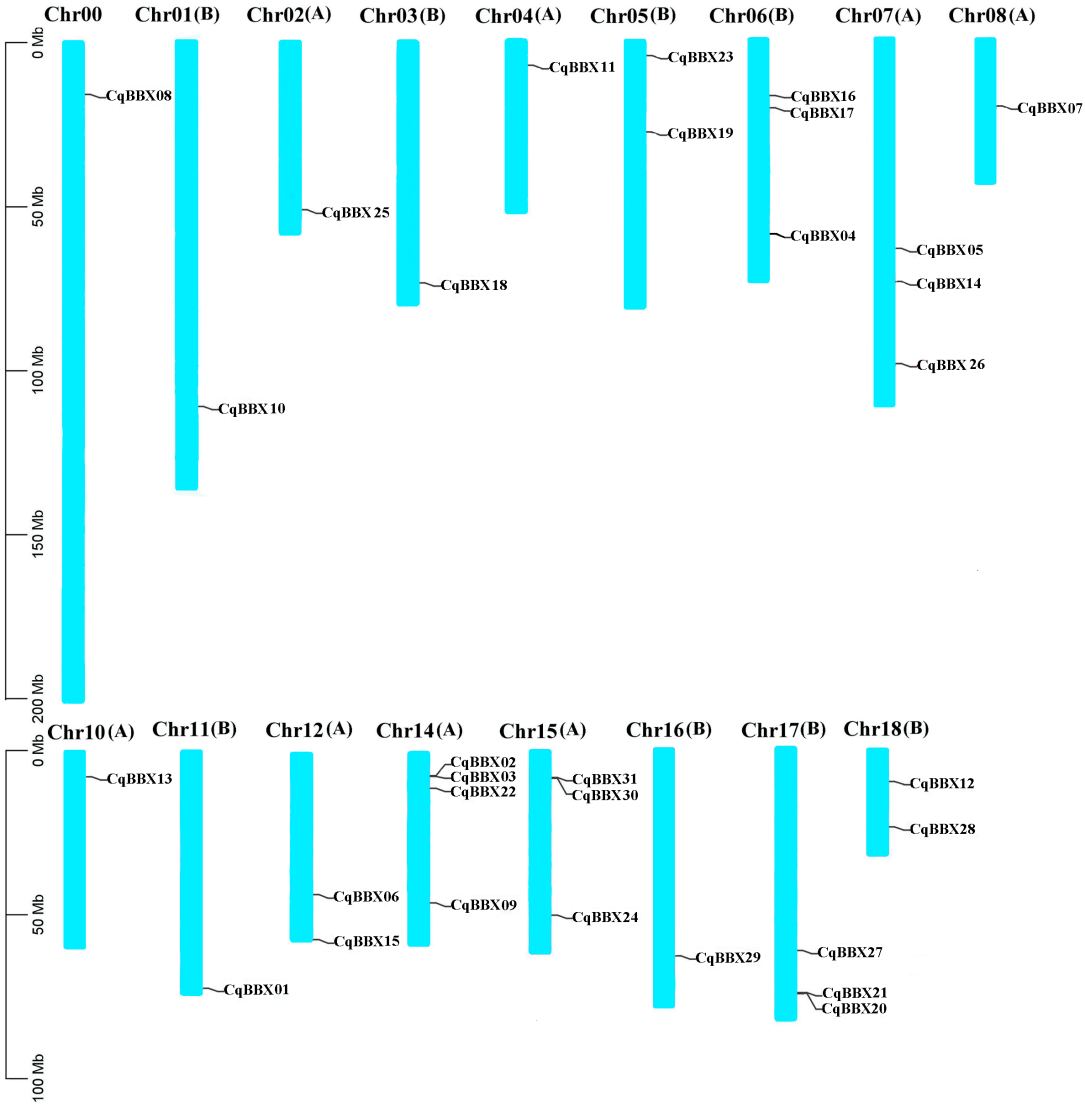
Chromosome localization of BBX gene in Quinoa genome. The scale on the left is based on megabytes (Mb). The number of chromosomes is indicated at the top of each column. Chromosome number suffixes A and B denote subgenome A and B in quinoa genome, respectively.

Based on the quinoa genome annotation file (Cq_PI614886_gene_V1_pseudomolecule.gff), the analysis results showed (Fig. 1) that 31 *CqBBX* genes were unevenly distributed on 17 chromosomes, of which *CqBBX08*, *CqBBX10*, *CqBBX25*, *CqBBX18*, *CqBBX11*, *CqBBX07*, *CqBBX13*, *CqBBX01*, and *CqBBX29* were distributed in Chr00, Chr01 (B), Chr02 (A), Chr03 (B), Chr04 (A), Chr08 (A), Chr10 (A), Chr11 (B) and Chr16 (B), *CqBBX23* and *CqBBX19* were distributed on Chr05 (B), *CqBBX16*, *CqBBX17*, and *CqBBX04* were distributed on Chr06 (B), *CqBBX05*, *CqBBX14*, and *CqBBX26* were distributed on Chr07 (A), *CqBBX06* and *CqBBX15* were distributed on Chr12 (A), *CqBBX02*, *CqBBX03*, *CqBBX22*, and *CqBBX09* were distributed on Chr14 (A), *CqBBX31*, *CqBBX30*, and *CqBBX24* were distributed on Chr15 (A), *CqBBX27*, *CqBBX21*, and *CqBBX20* were distributed on Chr17 (B), and *CqBBX12* and *CqBBX28* were distributed on Chr18 (B). In addition, most of the *CqBBX* genes were localized in the regions at both ends of the chromosome.

In the process of gene evolution and differentiation, gene replication plays an important role in gene expansion and functional differentiation of genes. By assessing the gene replication events of quinoa’s BBX, it was found that a total of 14 pairs of fragment copy events were generated (*CqBBX01*/*CqBBX05*, *CqBBX02*/*CqBBX15*, *CqBBX03*/*CqBBX16*, *CqBBX04*/*CqBBX09*, *CqBBX06*/*CqBBX19*, *CqBBX07*/*CqBBX29*, *CqBBX10*/*CqBBX11*, *CqBBX13*/*CqBBX18*, *CqBBX14*/*CqBBX27*, *CqBBX17*/*CqBBX22*, *CqBBX20*/*CqBBX30*, *CqBBX21*/*CqBBX31*, and *CqBBX26*/*CqBBX28*) (Table 3). This result suggested that fragment replication events may be important to the expansion of the BBX TF family in quinoa. In addition, we also calculated the Ka/Ks values of fragment gene pairs, and the results showed that all Ka/Ks values were less than 1, indicating that the *CqBBX* genes evolved mainly under the influence of purification selection.

**Table 3:** The Ka/Ks ratios and date of duplication for duplicate *CqBBX* genes.

Duplicated *CqBBX* gene1	Duplicated *CqBBX* gene2	Ka	Ks	Ka/Ks	MYA	Selective pressure	Duplicate type
CqBBX01	CqBBX05	0.011	0.144	0.074	9.602	Purifying selection	Segmental
CqBBX02	CqBBX15	0.009	0.131	0.067	8.711	Purifying selection	Segmental
CqBBX03	CqBBX16	0.020	0.149	0.136	9.922	Purifying selection	Segmental
CqBBX04	CqBBX09	0.045	0.177	0.256	11.818	Purifying selection	Segmental
CqBBX06	CqBBX19	0.013	0.148	0.086	9.871	Purifying selection	Segmental
CqBBX07	CqBBX29	0.024	0.151	0.157	10.077	Purifying selection	Segmental
CqBBX10	CqBBX11	0.007	0.094	0.077	6.278	Purifying selection	Segmental
CqBBX12	CqBBX24	0.018	0.099	0.184	6.625	Purifying selection	Segmental
CqBBX13	CqBBX18	0.045	0.151	0.295	10.097	Purifying selection	Segmental
CqBBX14	CqBBX27	0.027	0.055	0.490	3.645	Purifying selection	Segmental
CqBBX17	CqBBX22	0.042	0.109	0.387	7.249	Purifying selection	Segmental
CqBBX20	CqBBX30	0.007	0.056	0.129	3.705	Purifying selection	Segmental
CqBBX21	CqBBX31	0.015	0.110	0.133	7.347	Purifying selection	Segmental
CqBBX26	CqBBX28	0.023	0.141	0.160	9.407	Purifying selection	Segmental

### Phylogenetic analysis

**Figure 2:**
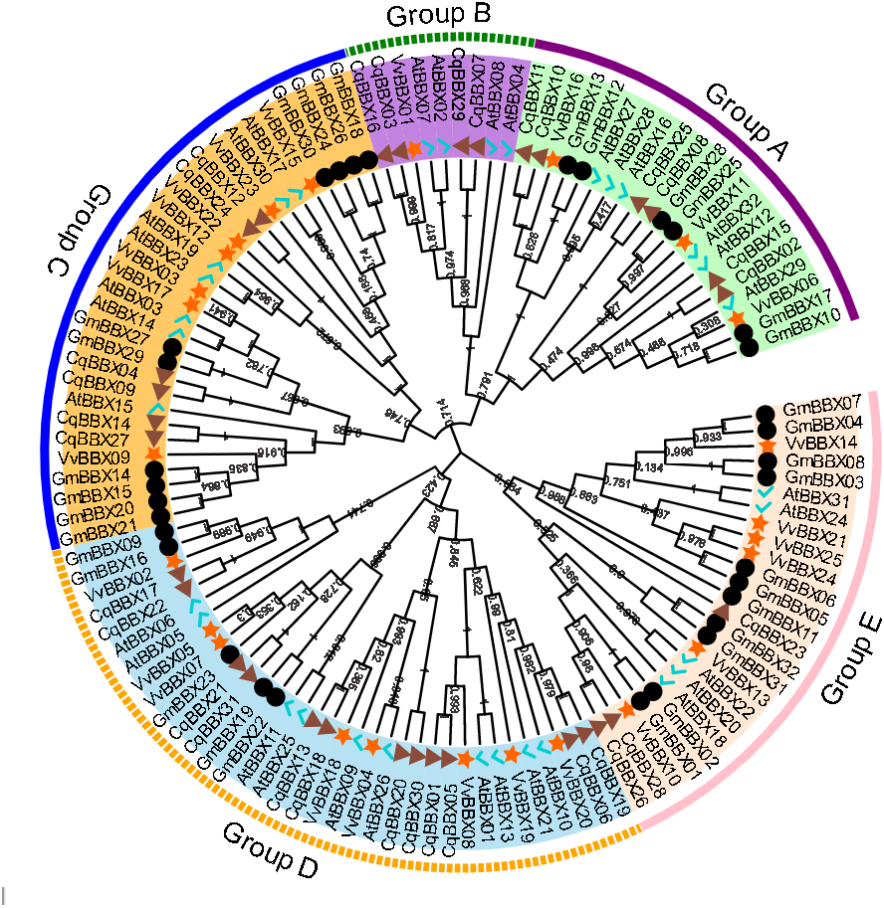
Phylogenetic analysis of BBX members from *Chenopodium quinoa*, *Glycine max*, *Vitis Vinifera*, and *A. thaliana*. The phylogenetic tree was constructed using the maximum-likelihood method in MEGA 11. Bootstrap values from 1,000 replicates were indicated at each branch. The BBX proteins from *Chenopodium quinoa*, *Glycine max*, *Vitis Vinifera*, and *A. thaliana* were marked with a triangle, circle, star, and check, respectively. Gene subfamilies were indicated with different colors and were classified into five subfamilies: Group A, Group B, Group C, Group D, and Group E.

In order to explore the evolutionary relationships of the BBX gene family of quinoa, the full-length protein sequences of 32 soybeans, 25 grapes, 32 *A. thaliana*, and quinoa BBX genes were used to construct a neighboring system evolutionary tree (Fig. 2, Table S2), and SMART was used to predict the conserved domain of each CqBBX (Fig. S1). The results showed that among the 31 CqBBX proteins, nine CqBBX contained two B-box domains and a conserved CCT protein domain. The five CqBBX contain a B-box and a CCT protein domain. Nine CqBBX contained two B-box domains, and eight CqBBX contained only one B-box domain. According to phylogeny, the 31 *CqBBX* and soybean, grape, and *A. thaliana* homologs were divided into five subfamilies (GroupA, GroupB, GroupC, GroupD, and GroupE). The A subfamily had six *CqBBX* members, and they all contained two B-box domains and one CCT protein domain. The B subfamily had four *CqBBX* members, and they all contained one B-box domain and one CCT protein domain. There were six *CqBBX* members in subfamily C, among which *CqBBX27*, *12*, and *24* all contained two B-box domains and one CCT protein domain, *CqBBX04* contained one B-box domain and one CCT domain, *CqBBX14* contained two B-box domains, and *CqBBX09* contained only one B-box domain. There were twelve *CqBBX* members in subfamily D, except for *CqBBX13*, *CqBBX20*, *CqBBX30*, and *CqBBX31*, which only contained one B-box domain, the other eight *CqBBX* members contained two B-box domains. The E subfamily had three *CqBBX* members, and they all contained one B-box domain.

### Gene structure and conserved motifs of BBX family members of quinoa

**Figure 3:**
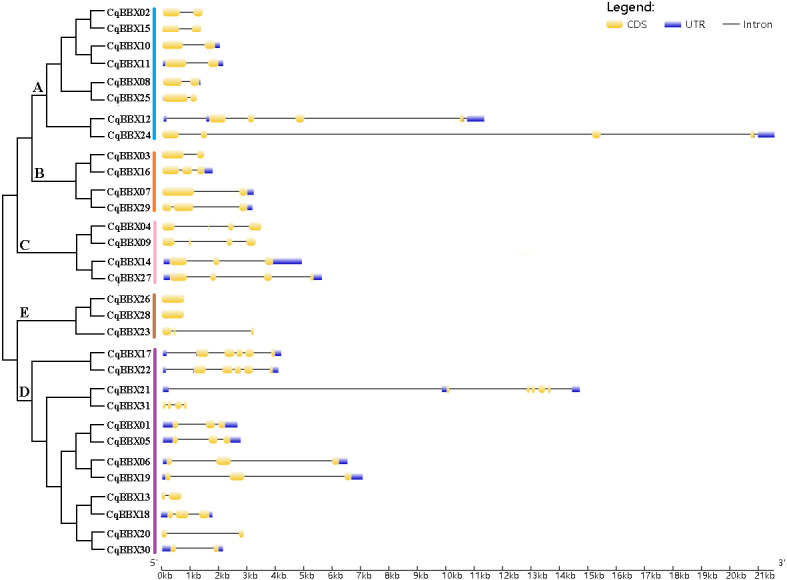
The *CqBBX* gene structures. The black line and yellow box respectively represent introns and exons. The blue box represents a 3′ or 5′ untranslated region. The lengths of exons and introns are indicated by the scale bar.

To further understand the function of the *CqBBX* gene family, structural analysis of the *CqBBX* genes was performed using the CDS sequence (Fig. 3). The results showed that the CqBBX proteins were divided into five subfamilies. Except for *CqBBX12* and *CqBBX24*, the subgroups of other CqBBX proteins were consistent with the results of the phylogenetic analysis. Moreover, we found that genes on the same branch were highly similar in structure. For example, *CqBBX02* and *CqBBX15*, *CqBBX17* and *CqBBX22*, *CqBBX26* and *CqBBX28*, as well as *CqBBX01* and *CqBBX05*. These results suggested that the genetic structure of the *CqBBX* gene family was closely related to its evolution. In addition, we found that in the *CqBBX* gene family, except for *CqBBX26* and *CqBBX28*, which had no introns, the number of introns of other members was between one and six. Most of them contained only one intron, such as *CqBBX13*, *CqBBX20*, *CqBBX30*, *CqBBX03*, *CqBBX07*, *CqBBX02*, *CqBBX15*, *CqBBX10*, *CqBBX11*, *CqBBX08* and *CqBBX25*, a total of 11. In addition, there were 11 genes without 3′ or 5′ untranslated regions, namely *CqBBX26*, *CqBBX28*, *CqBBX23*, *CqBBX04*, *CqBBX09*, *CqBBX13*, *CqBBX20*, *CqBBX03*, *CqBBX31*, *CqBBX02,* and *CqBBX15*.

**Figure 4:**
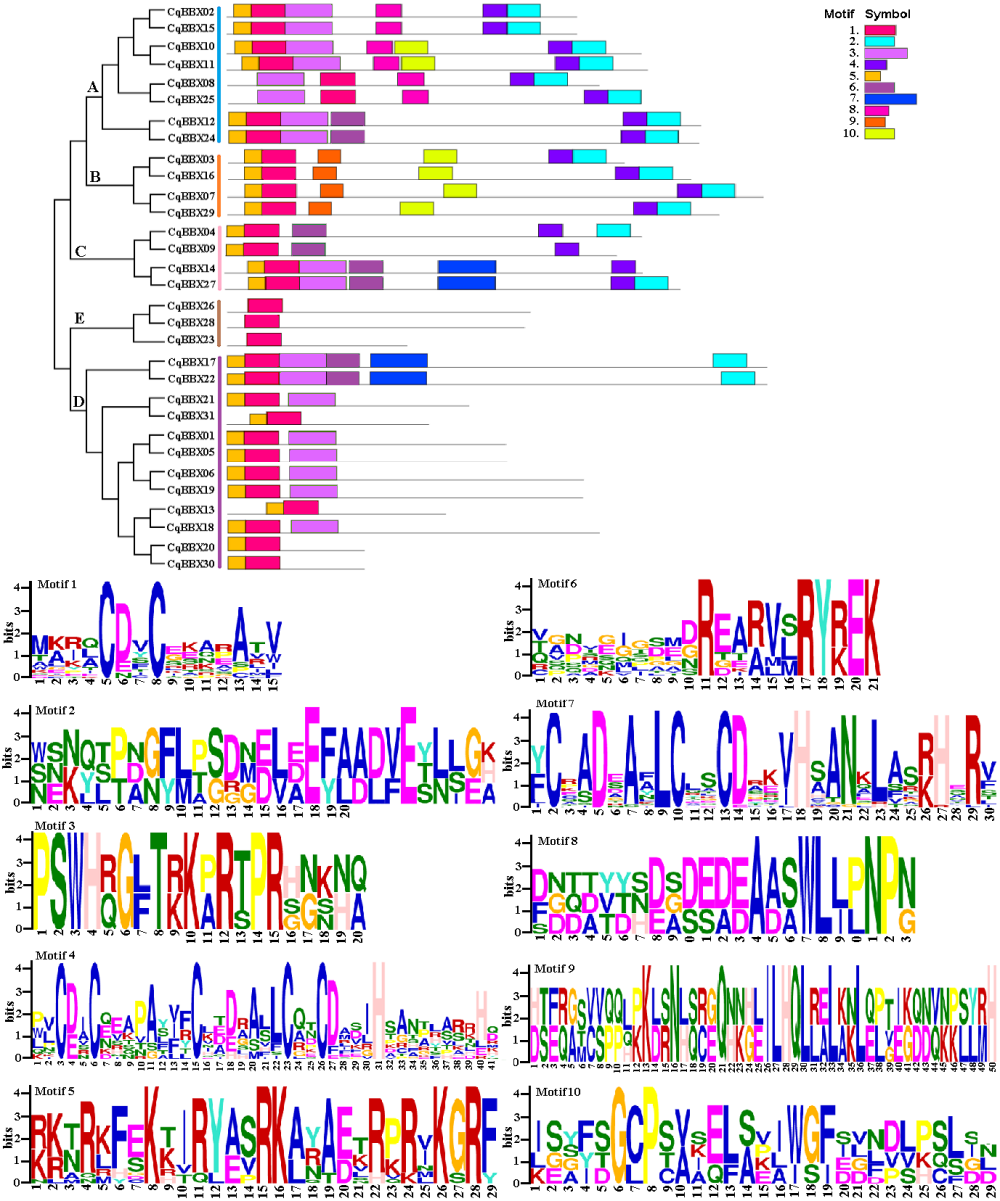
The CqBBX conserved motifs and 10 protein domains. Each colored box represents a conservative region.

To further explore the structural diversity of CqBBX proteins, the composition and number of conserved motifs of 31 CqBBX proteins were predicted on the MEME online website, and 10 motifs were identified and named motif 1 to motif 10 in turn (Fig. 4). Combined analysis with the domains of 31 CqBBX proteins (Fig. S1) revealed that both motif 1 and motif 3 were associated with the B-BOX domain, and the CCT domain was probably formed by motif 2. In addition, we found that all members of subgroups B and E had the same composition and number of conserved motifs, while members of the other three subgroups had different conserved motifs. But there are exceptions. For example, in subgroup C, *CqBBX14* and *CqBBX27* both had motif 3 and motif 7, while *CqBBX04* and *CqBBX09* did not, but *CqBBX04* and *CqBBX27* also had motif 2, while *CqBBX09* and *CqBBX14* did not. And the same thing happened in subgroup D. The above results showed that the motif and exon/intron structure of different groups were different, but they were highly conserved on the same branch. The results showed clear conservation, laying the foundation for functional conservation and providing guidance for subsequent functional studies.

### Promoter cis-acting element analysis of quinoa BBX gene family

**Figure 5:**
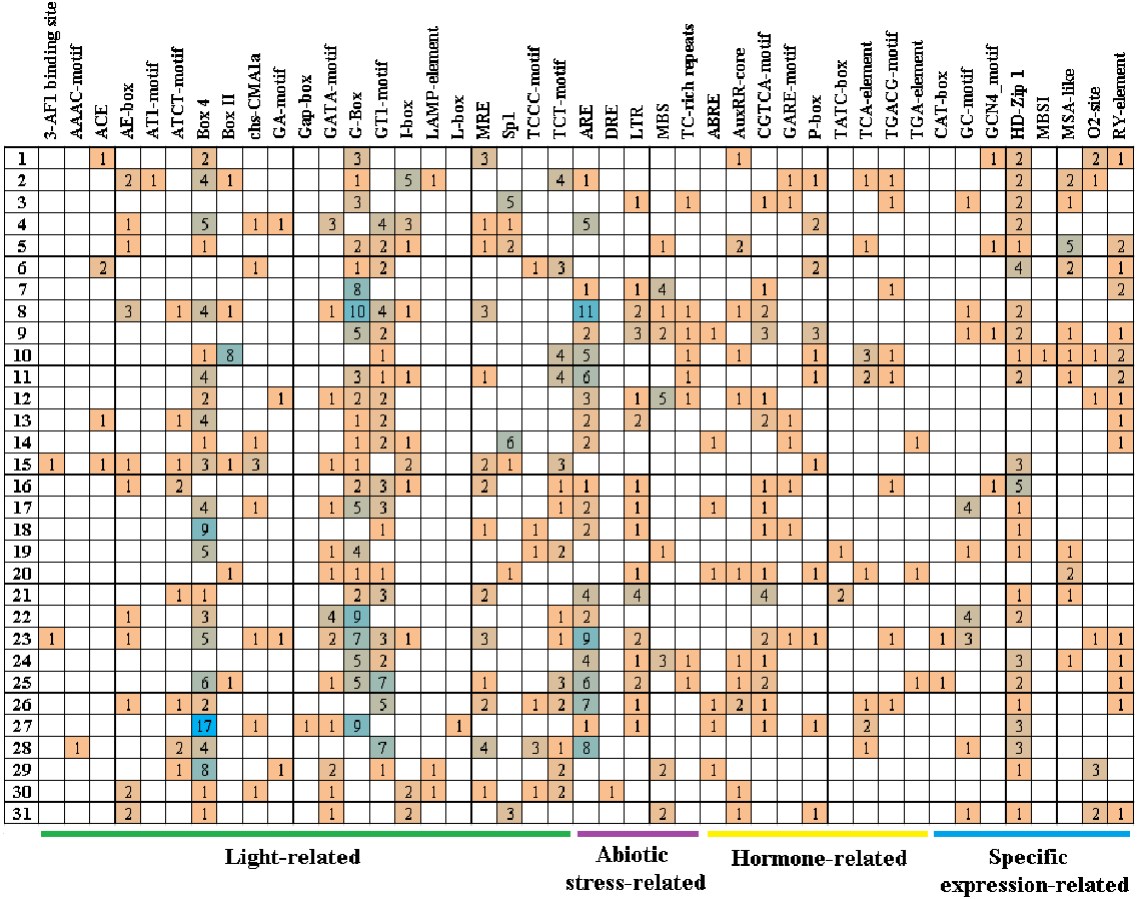
The cis-elements that respond to light, plant hormones, tissue specific expression and abiotic stress signals within the *CqBBX* gene promoters. On the left side of the 1–31 respectively represent *CqBBX01* to *CqBBX31*. The numbers in the figure represent the number of cis-acting elements.

*Cis*-acting elements play critical roles in regulatory networks controlling plant growth and development and are closely related to determining the tissue-specific or stress-response expression profile of genes. In order to analyze the response mechanism of the quinoa BBX TF family to light, hormone, tissue-specific expression, and abiotic stress, the acting elements on the promoter sequence of 2,000 bp upstream of the family gene starting codon were analyzed using the PlantCARE online website. The results showed that a total of 43 cis-elements were screened out (Fig. 5, Table S3). Its 43 cis-elements were divided into four groups, of which the light response involved 21 cis-elements, including 3-AF1 binding site, AAAC-motif, ACE, AE-box, AT1-motif, ATCT-motif, Box 4, Box II, CHS-CMA1a, GA-motif, Gap-box, GATA-motif, G-box, GT1-motif, I-box, LAMP-element, L-box, MRE, Sp1, TCCC-motif, and TCT-motif, there were 100 G-boxes, which accounted for the largest part of the light response element, accounting for 22.5%. 5 cis-elements were associated with abiotic stress, these included AER (anaerobic induction), DRE (dehydration, low-temperature, salt stresses), TC-rich repeats (defense and stress responsiveness), LTR (low-temperature), and MBS (drought-inducibility). Nine cis-elements were involved in the hormone response, namely ABRE, AuxRR-core, CGTCA-motif, GARE-motif, P-box, TATC-box, TCA-element, TGACG-motif, and TGA-element, among them, there were 84 cis-elements ABRE involved in the abscisic acid reaction, accounting for the largest proportion of 44.0% in this category. There were eight cis-elements related to tissue-specific expressions, namely CAT-box, GC-motif, GCN4_motif, HD-Zip 1, MBSI, MSA-like, O2-site, and RY-element, among them, the cis-element O2-site, which was involved in the metabolism regulation of corn protein, accounted for the largest proportion, accounting for 25.8%, followed by the abscisic acid response element CAT-box, accounting for 22.7%. These results suggested that light, hormones, and stress may affect the expression level of the *CqBBX* genes. In addition, the BBX genes may respond to abiotic stress and improve the abiotic stress response.

### Protein interaction prediction of the BBX gene family of quinoa

**Figure 6:**
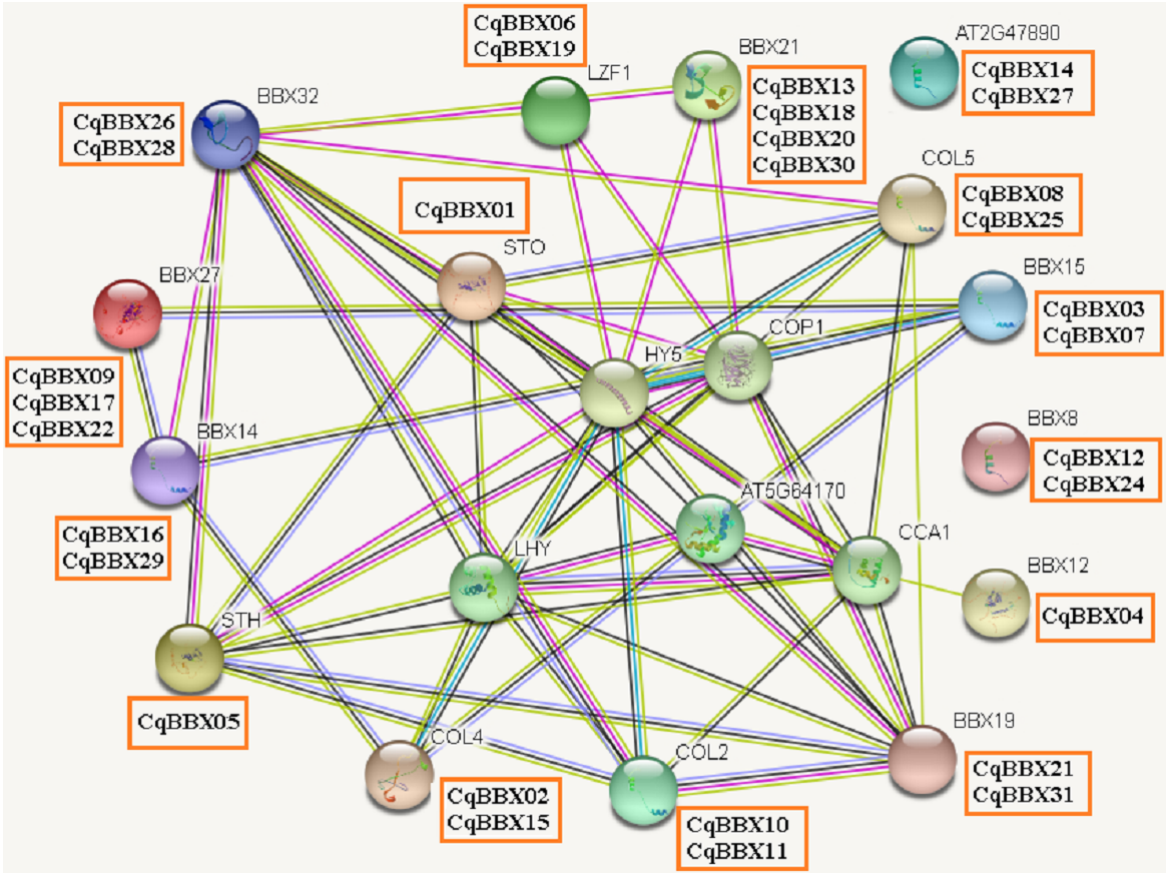
The CqBBX protein interaction network analysis. The orange boxes and spheres indicate quinoa BBX protein and *A. thaliana* BBX protein with which it interacts, respectively.

It was well known that most proteins in plants interact with each other and were involved in plant growth development and stress. To better understand the molecular mechanisms of CqBBX, an interaction network between CqBBX proteins and *A. thaliana* proteins was constructed (Fig. 6). The results showed that a total of 20 *A. thaliana* proteins and 31 CqBBX proteins formed a protein interaction relationship. Studies had shown that STO was a salt-tolerant gene whose protein negatively regulates photosensitive pigment and blue light signaling pathways, and STH was similar to STO and interacted with CONSTITUTIVELY PHOTOMORPHOGENIC1 (COP1) protein, a core suppressor of light morphology (Jiang et al., 2012; Sarmiento, 2013). *CqBBX01* and *CqBBX05* interact with STO and STH, indicating that *CqBBX01* and *CqBBX05* may also have similar functions. *AtBBX21* played an important role in the establishment of light morphology, and it was manifested as hypocotyl elongation in both red, far-red, and blue light, while LZF1 (*AtBBX22*) protein accumulated to higher levels under short sunshine conditions and had the function of inhibiting hypocotyl elongation. COP1 is an E3 ubiquitin ligase, and many photomorphogenesis promoters can be degraded by COP1, thereby inhibiting photomorphogenesis (Lau & Deng, 2012; Huang, Ouyang & Deng, 2014). HY5 is a bZIP transcription factor that positively regulates light signaling (Oyama, Shimura & Okada, 1997). The main reason that HY5 is ubiquitinated and degraded by COP1 in the dark but not in the light is that light mediates the inhibition of COP1 activity through multiple mechanisms (Osterlund et al., 2000; Hoecker, 2017; Podolec & Ulm, 2018). In addition, COP1 mediated the degradation of LZF1 in the dark, and HY5 contributed to the degradation of LZF1 in light. The constitutive photomorphogenic development of *cop1* mutants in cop1BBX22ox plants was enhanced, and the hypocotyl was shortened, high anthocyanin accumulation and light-responsive gene expression were enhanced (Chang, Maloof & Wu, 2011). Since COP1 interacts with STO, STH, LZF1, HY5, and other proteins, we predicted that some BBX proteins in quinoa (*CqBBX01*, *CqBBX05*, *CqBBX06*, and *CqBBX19*, *etc.*) also interact with COP1. These results suggest that these BBX proteins in quinoa, like COP1 protein, play a very important role in the light growth of plants. In addition, *AtBBX21* was a positive regulator of anthocyanin synthesis (Crocco et al., 2018), while *AtBBX32* inhibited the biosynthesis and accumulation of anthocyanins (Preuss et al., 2012), and the functions of *CqBBX13*, *CqBBX18*, *CqBBX20*, and *CqBBX30* may be relative to *CqBBX26* and *CqBBX28*. In *A. thaliana*, the photoperiod flowering pathway was controlled by a set of regulators, including CONSTANS (CO), in addition, *A. thaliana* had a family of genes homologous to CO, called CO-LIKE (COL), and the findings suggested that the constitutive expression of COL5 partially inhibits the late flowering phenotype of commonly mutant plants (Hassidim et al., 2009). Some scholars also investigated the role of CONSTANS-LIKE 4 (COL4) in *A. thaliana*, and its results showed that a decrease in COL4 expression levels led to an increase in FT and APETALA 1 (AP1) expression and accelerated flowering, while an increase in COL4 expression led to a delay in flowering. Therefore, we concluded that the two genes *CqBBX02* and *CqBBX15* may also be related to the flowering time of quinoa (Steinbach, 2019).

### The transcripts amounts of *CqBBXs* under different tissues and abiotic stresses analysis based on high-throughput sequencing

**Figure 7:**
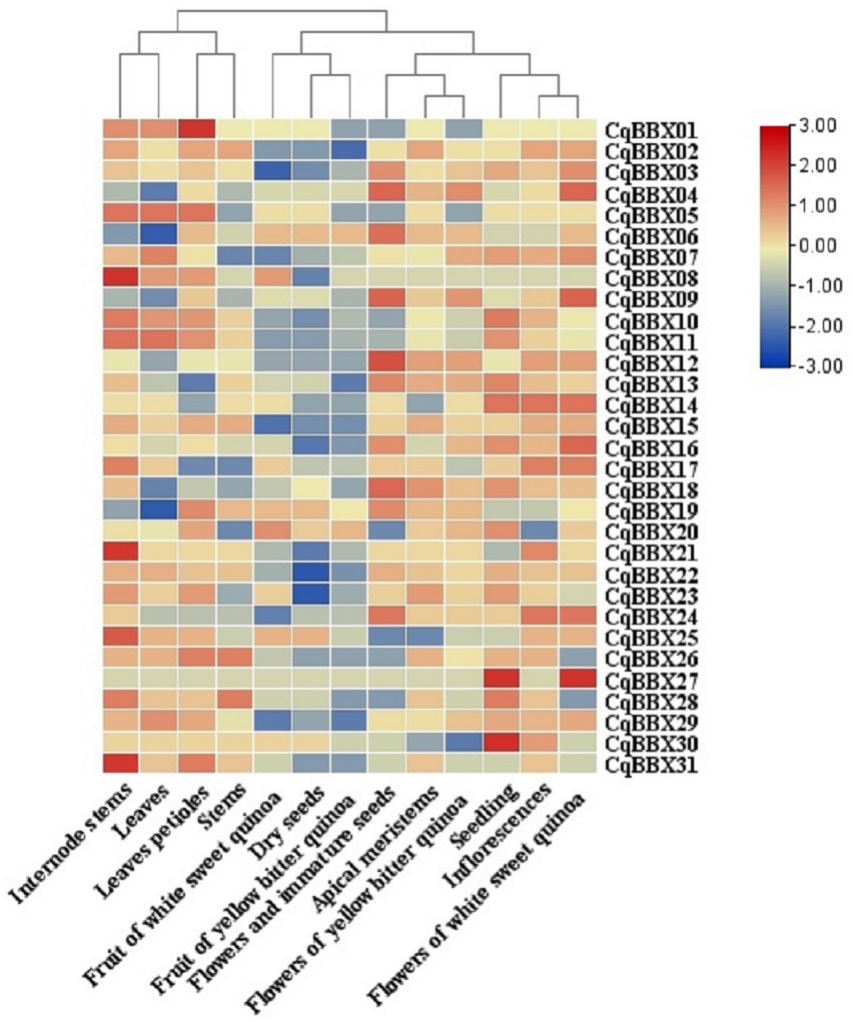
Transcripts amounts of the *CqBBX* in different tissues. Tissues include apical meristems, flowers, and immature seeds, stems, inflorescences, petioles, fruits of yellow bitter quinoa, fruits of white sweet quinoa, dried seeds, flowers of white sweet quinoa, flowers of yellow bitter quinoa, internodal stems, leaves, and seedlings. The bars in the lower right corner represent log_2_ FPKM values, and different colors indicate different levels of transcripts. Red indicates relatively high transcripts amounts and blue indicates relatively low transcripts amounts.

In order to study the specific expression of *CqBBXs* in different tissues, we used publicly available RNA-seq data to study *CqBBXs* transcripts amounts in 13 different tissues including apical meristem, flowers, and immature seeds, leaves petioles, stems, internode stems, seedling, inflorescences, leaves, dry seeds, flowers of white sweet quinoa, fruit of white sweet quinoa, flowers of yellow bitter quinoa and fruit of yellow bitter quinoa. Based on hierarchical clustering, the 13 tissues were divided into two groups (Fig. 7, Table S4). The results showed that in the first group of four tissues (internode stem, leaf, petiole, and stem), the transcripts amounts of the *CqBBX* genes were higher in the internode stem than in the other three tissues, among which *CqBBX08*, *CqBBX21*, and *CqBBX31* were the most prominent, and the transcripts amounts of *CqBBX01* was the most prominent in the petiole. The *CqBBX* gene in the remaining two tissues showed low or even no transcripts amounts. In the second group of nine tissues (white sweet quinoa fruit, dry seeds, yellow bitter quinoa fruit, flowers, and immature seeds, apical meristem, yellow bitter quinoa flowers, seedlings, inflorescences, and flowers of white sweet quinoa), several genes showed high transcripts amounts in three tissues: flowers and immature seeds, seedlings, and white sweet quinoa flowers. For example, *CqBBX04*, *CqBBX06*, *CqBBX09*, and *CqBBX12* showed high transcripts amounts in flowers and immature seeds, while *CqBBX27* and *CqBBX30* showed high transcripts amounts in seedlings. *CqBBX27*, *CqBBX09*, and *CqBBX04* showed high transcripts amounts in white sweet quinoa flowers. These results suggested that *CqBBXs* may play a different role in the growth and development of quinoa.

**Figure 8:**
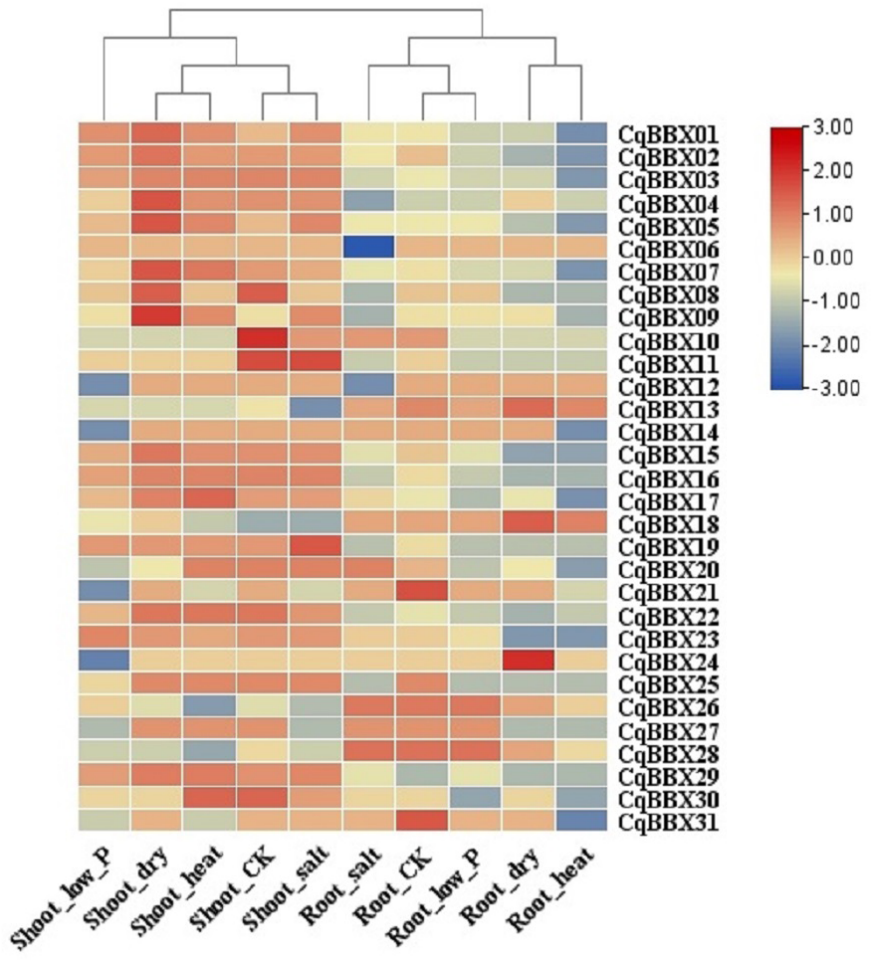
Transcripts amounts of *CqBBX* in roots and shoots of quinoa under drought, heat, low-Pi, and salt stress. The bars in the lower right corner represent log_2_ FPKM values, and different colors indicate different levels of transcripts. Red indicates relatively high transcripts amounts and blue indicates relatively low transcripts amounts.

In order to study the transcripts amounts of *CqBBXs* under abiotic stress, 30 d leaf-aged quinoa seedlings were treated with drought, heat, low-Pi, and salt stress. The transcripts amounts of the *CqBBX* genes were obtained by RNAseq (Fig. 8, Table S4). The results showed that in the roots, the gene transcripts amounts of *CqBBX26* and *CqBBX28* were induced under salt and low-Pi stress. In the control, the transcripts amounts of the *CqBBX21* gene were induced, followed by *CqBBX31*; the gene transcripts amounts of *CqBBX24* were induced under drought stress, followed by *CqBBX13* and *CqBBX18*. In shoots, the gene transcripts amounts of *CqBBX01*, *CqBBX02*, *CqBBX03*, *CqBBX04*, *CqBBX05*, *CqBBX07*, *CqBBX08*, and *CqBBX09* were induced under drought, heat, and salt stress. In the control, *CqBBX10* gene transcripts amounts were induced, followed by *CqBBX08* and *CqBBX11*; in addition, the gene transcripts amounts of *CqBBX11* were induced under salt stress, followed by *CqBBX19*. All of the above results suggested that *CqBBXs* had a potential role in improving the tolerance of quinoa to water stress.

### Quantitative reverse transcription PCR

**Figure 9:**
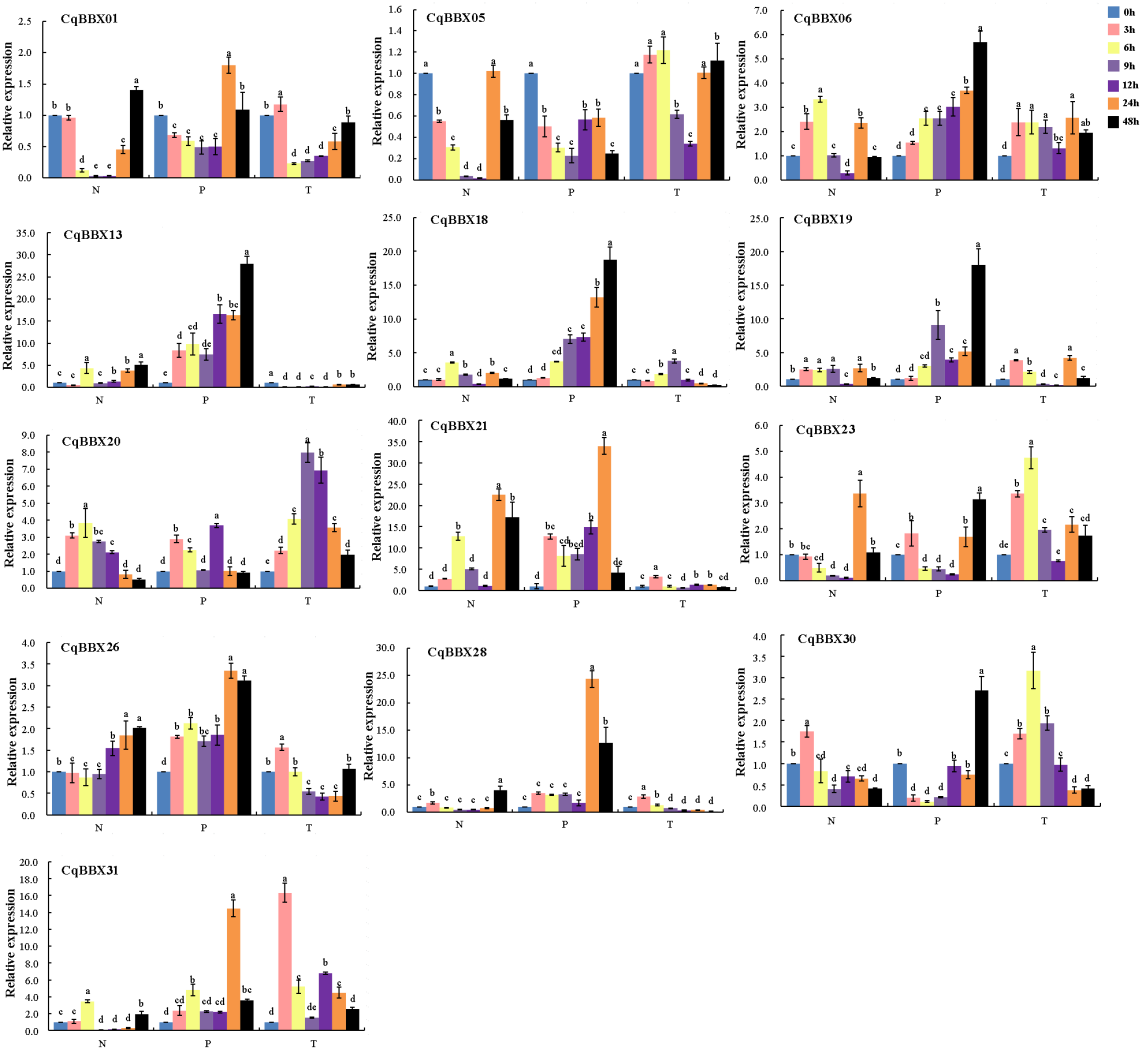
The transcripts amounts of *CqBBX* under salt (N), drought (P) and low-temperature (T) stress was detected by qRT PCR. Blue is 0 h, pink is 3 h, yellow is 6 h, lilac is 9 h, purple is 12 h, orange is 24 h, and black is 48 h. Taking 0 h as the control, the gene transcripts amounts was 1. The character at the top of the error bar represents standard errors among three replicates, and different letters indicate significant differences among treatments (*P* < 0.05).

NaCl, PEG, and low-temperature treated plant leaves were used for qRT-PCR to evaluate gene transcripts amounts (Fig. 9, Table S5). The results showed that compared with the control, the transcripts amounts of *CqBBX* genes were misregulated in both directions up and down-regulated in different time periods under NaCl stress. For example, *CqBBX06, CqBBX19, CqBBX20, CqBBX28*, and *CqBBX30* genes were significantly up-regulated under salt stress for 3 h. At the 6 h hour, *CqBBX06*, *CqBBX13*, *CqBBX18*, *CqBBX20*, *CqBBX21*, and *CqBBX31* genes were significantly up-regulated, and these genes basically showed a downregulation trend at 9 and 12 h. However, at the 24 h under NaCl stress, *CqBBX05*, *CqBBX06*, *CqBBX13*, *CqBBX18*, *CqBBX21*, and *CqBBX23* genes were significantly up-regulated, and the transcripts amounts of *CqBBX21* gene at the highest level reached 22.47, which was 21 times higher than that at the lowest level. These results indicate that different BBX genes respond differently to NaCl stress at different treatment times.

Compared with the control, except for *CqBBX05*, the remaining *CqBBX* genes under PEG stress showed up-regulation. Moreover, with the increase in treatment time, the transcripts amounts of *CqBBX06*, *CqBBX13*, and *CqBBX18* were up-regulation. At 24 h under PEG stress, *CqBBX01*, *CqBBX21*, *CqBBX26*, *CqBBX28*, and *CqBBX31* were significantly up-regulated, and the most significant was *CqBBX21*, whose transcripts amounts were 34.01. At 48 h, *CqBBX06*, *CqBBX13*, *CqBBX18*, *CqBBX19*, *CqBBX23*, and *CqBBX30* were significantly up-regulated, and the most significant was *CqBBX13*, whose transcripts amounts were 27.79. These results suggest that *CqBBX* genes may be affected by drought stress.

Compared with the control, *CqBBX01*, *CqBBX06*, *CqBBX19*, *CqBBX20*, *CqBBX21*, *CqBBX23*, *CqBBX26*, *CqBBX28*, *CqBBX30*, and *CqBBX31* genes were significantly up-regulated at low-temperature stress for 3 h, among which *CqBBX31* was the most significant, its transcripts amounts was 16.33. After 6 h of low-temperature stress, *CqBBX01*, *CqBBX05*, *CqBBX13*, *CqBBX21,* and *CqBBX26* genes were significantly upregulated, among which *CqBBX23* was the most significant, and its transcripts amounts were 4.75. In addition, except for *CqBBX06*, *CqBBX18*, *CqBBX20*, *CqBBX21*, *CqBBX23*, *CqBBX30,* and *CqBBX31*, other genes were significantly down-regulated after 9 and 12 h of low-temperature stress. The *CqBBX06*, *CqBBX13*, *CqBBX19*, *CqBBX20*, *CqBBX23,* and *CqBBX31* genes were significantly up-regulated after 24 h of low-temperature stress. *CqBBX05*, *CqBBX06*, *CqBBX20,* and *CqBBX23* genes were significantly upregulated after 48 h of low-temperature stress. In general, although gene regulation was significant compared with control, the amounts of transcripts were not high under low-temperature stress.

## Discussion

BBX is an important zinc finger protein that plays a key role in plant growth and development and in response to environmental changes, and it is a TFs protein unique to plants, containing one or two B-box domains and CCT protein domains (Khanna et al., 2009; Gangappa & Botto, 2014). To date, 32 BBX members have been identified in *A. thaliana* (Lyu, Li & Li, 2020), 30 BBX members in rice (Huang et al., 2012), 29 BBX members in tomatoes (Chu et al., 2016), 30 BBX members in potatoes (Talar et al., 2017), and 28 BBX members in pears (Cao et al., 2017). However, information on BBX family members of quinoa has not been reported. Therefore, genome-wide analysis of the BBX gene in quinoa will lay the foundation for further functional studies of this gene family.

We identified 31 CqBBX members, most of whom were weakly acidic proteins and all family members were hydrophilic proteins, which were similar to the physicochemical properties of the BBX proteins in petunias (Wen et al., 2020) and grape (Wei et al., 2020), indicating that the BBX gene was highly conserved across species. It is known that in *A. thaliana*, the BBX family is divided into five subgroups based on the number of B-box domains and the presence of CCT protein domains. The first and second subgroups have a total of 13 members, containing two B-box domains and a CCT protein domain. The third subgroup has four members and contains a B-box domain and a CCT protein domain, while the fourth subgroup has eight members and contains two B-box domains, and the fifth subgroup has seven members and only one B-box domain. In this study, ML evolutionary tree was constructed and divided into five subgroups based on BBX protein sequences of *A. thaliana*, grapevine, soybean, and quinoa. However, unlike *A. thaliana*, quinoa BBX cannot cluster each subgroup together according to its domain, and within each subfamily, the *AtBBX* differs in domain type and number with the *CqBBX*. For example, in GroupA, where the *CqBBX* members have two B-box domains and one CCT protein domain, *AtBBX27*, *28*, *29*, and *30* contain only one B-box domain, suggesting that the BBX proteins of quinoa and *A. thaliana* may have different biological functions in the same branch, and this is also found in pineapple (Ouyang et al., 2022) and petunia (Wen et al., 2020). In addition, this was also the case in rice (Huang et al., 2012) and tomato (Chu et al., 2016), where subgroups could not be divided according to the domain. For example, in rice, *OsBBXs* with two B-box domains and one CCT protein domain and one B-box domain and one CCT protein domain cannot be divided into different subfamilies. This may be because some B-box2 may have been removed during evolution. Some scholars have found that there are very large differences between the gene structure and molecular properties of the BBX genes in plants through an in-depth understanding of the evolution and expansion of the BBX genes family, which indicates the wide diversity of BBX family members (Yu et al., 2022).

Gene replication events play a crucial role in the evolutionary process of plants and in the expansion of gene family members (Cannon et al., 2004). In order to further elucidate the amplification mechanism of the *CqBBX* gene family in quinoa, the *CqBBX* gene replication event was analyzed. The results showed that there were 14 pairs of genes involved in fragment replication events (*CqBBX01/CqBBX05*, *CqBBX02/CqBBX15*, *CqBBX03/CqBBX16*, *CqBBX04/CqBBX09*, *CqBBX06/CqBBX19*, *CqBBX07/CqBBX29*, *CqBBX10/CqBBX11*, *CqBBX12/CqBBX24*, *CqBBX13/CqBBX18*, *CqBBX14/CqBBX27*, *CqBBX17/CqBBX22*, *CqBBX20/CqBBX30*, *CqBBX21/CqBBX31* and *CqBBX26/CqBBX28*) (Table 4), indicating that fragments replication were more common in *CqBBX* genes. The same underlying mechanisms for gene family evolution have also been identified with the BBX gene family of grapes (Wei et al., 2020). In addition, the Ka/Ks values of fragment gene pairs were calculated. Generally, Ka/Ks ratio greater than 1 signifies positive selection with accelerated evolution, Ka/Ks ratio equal to 1 represents neutral selection, while less than 1 means stabilizes or negative selection. Remarkably, Ka/Ks ratios for all homologous gene pairs were less than 1, suggesting that these gene pairs underwent significant purification selections during their evolution. This was also consistent with the Ka/Ks values of fragment gene pairs in the grape BBX gene family (Wei et al., 2020).

The distribution of exons and introns in the genetic structure can further be shown to be the same as the groupings in the phylogenetic tree. In this study, the gene structure was divided into five subfamilies, each of which had individual genes without 3′ or 5′ untranslated regions. In addition, most *CqBBXs* clustered in the same subfamily showed different exon-intron structures, but most *CqBBX* genes on the same branch were highly similar in structure. These results suggested that gene replication may have occurred during gene evolution, resulting in a diversity of gene structures.

In this study, we detected a number of cis-acting elements in the promoter region of *CqBBX* and ultimately screened for four classes of elements associated with light, hormone, tissue-specific expression, and abiotic stress. Among them, 21 elements were related to the light response, indicating that *CqBBX* played a role in light reactions. At the same time, nine hormone response elements were identified with the promoter of the quinoa BBX genes, and previous studies found that five hormone response elements were identified in the cotton BBX gene promoter (Feng et al., 2021), six cis-elements were involved to the hormonal response in the promoter of the dwarf morning glory BBX gene (Wen et al., 2020), and some response elements such as abscisic acid, ethylene, and gibberellin were also present in the promoter of the grape BBX gene (Wei et al., 2020). This suggests that the BBX gene had a potential role in hormonal responses. In addition, we found five cis-elements associated with abiotic stress. Previous studies have found that three drought-reactive cis-elements were detected in the promoter of *PhBBX20*, namely ABRE, STRE, and MBS (Wen et al., 2020). In tomatoes, 10 drought-regulated genes were also detected in 28 *SlBBXs*, and seven-tenths of *SlBBXs* were up-regulated after PEG treatment for 24 h (Chu et al., 2016). However, in pears, researchers have found that a total of 16 genes were regulated by drought, and 13 of them were up or down within 12 h of dehydration (Zou et al., 2018). We speculated that the timing of these reactions may be related to differences in species and treatment methods. Taken together, BBX played a key role in stress response, and *CqBBX* can be used to improve abiotic stress tolerance in quinoa.

Studies have shown that BBX had different modes of expression in different tissues (Chu et al., 2016; Huang et al., 2012; Zou et al., 2018). In our study, the number of transcripts of 31 members of the quinoa BBX gene family differed in different tissues of quinoa. For example, in the seven tissues of internodal stems, leaves, petioles, flowers, and immature seeds, seedlings, inflorescences, and flowers of white sweet quinoa, the transcripts amounts of 31 *CqBBX* genes were relatively high, but there were also individual genes with low transcripts amounts. The transcripts amounts of 31 *CqBBX* in the three tissues of white sweet quinoa fruit, dried seed, and yellow bitter quinoa are relatively low, and even many *CqBBX* genes did not respond in these tissues. Previous studies have shown that the BBX genes played a key role in regulating flowering, such as *AtBBX1*, *AtBBX4*, *AtBBX7*, and *AtBBX32* in *A. thaliana* (Putterill et al., 1995; Datta et al., 2006; Cheng & Wang, 2005; Tripathi et al., 2017). In our study, some cis-elements associated with flowering were identified in the *CqBBX* promoter region, such as the GCN4_motif required for endosperm expression. Corresponding *CqBBX* genes (such as *CqBBX04*, *CqBBX06*, *CqBBX09*, and *CqBBX12*) also had high transcripts amounts in flower organs, suggesting that these genes may play an important role in the formation of reproductive organs. Based on the analysis of transcriptome data, the results of this study showed that the transcripts amounts of *CqBBX* under different stresses in roots and young shoots were diverse and specific. Overall, the *CqBBX* family was widely induced or inhibited by abiotic adversity. In a study of 31 *CqBBX* genes, it was found that their transcripts amounts in various stresses in young shoots had an inducing effect, while in the roots the inhibition was greater than the inducing effect. However, under the low temperature and salt stress of shoots, there were also individual genes with low transcripts amounts, such as *CqBBX12*, *CqBBX14*, *CqBBX21,* and *CqBBX24*, which did not respond under low-temperature stress of shoots, and at the same time, *CqBBX13* did not respond under salt stress of shoots. In addition, we found that under the heat stress in the root, except for a few genes with low transcripts amounts, the rest of the genes were not responding.

In plants, many stress-related genes can produce stress responses that are regulated or mediated by various signaling pathways (Walther, Brunnemann & Selbig, 2007). The number of BBX gene families has been shown to play an active role in abiotic stress responses and is regulated by environmental signals (Huang et al., 2012; Liu et al., 2018). We found five cis-regulatory elements associated with abiotic stress in the *CqBBX* promoter region, such as DRE associated with water deficiency, hypothermia, and salt stress, LTR involved in cryogenic reactions, MBS associated with drought, TC-rich repeats, and ABRE involved in defense and stress responses. We also noted that these BBX genes contain at least one abiotic stress cis-element, suggesting that they may be helpful in responding to biological and abiotic stress, and there have been some studies on the BBX gene played a positive role in abiotic stress resistance (Chu et al., 2016; Wei et al., 2020), therefore, we thought that *CqBBXs* might respond to abiotic stress. In order to gain an in-depth understanding of the stress response mechanism of *CqBBX* genes, we selected 13 *CqBBX* genes to measure the relative expression of these genes under low temperature, drought, and salt stress conditions. The results showed that 13 *CqBBXs* were regulated by abiotic stresses, and their transcripts amounts were different for each stress. In our study, we identified 18 cis-elements associated with low temperatures, and all the other genes were up-regulated except *CqBBX13* under low-temperature stress, suggesting that these cis-elements may be positively correlated with the transcripts amounts of these genes. Studies have shown that in pears, most genes were up-regulated in cold conditions, except for *PbBBX18* down-regulated (Zou et al., 2018). We speculate that the BBX gene may function differently in different species. Under salt stress, some genes were up-regulated first and then down-regulated with the increase of treatment time, while others were down-regulated with the increase of treatment time, but these genes were suddenly up-regulated at 24 h of treatment. We speculate that salt stress at 24 h may be the highest point of transcripts amounts of the *CqBBX* genes. After PEG stress, most *CqBBXs* were up-regulated after drought stress, such as *CqBBX06*, *CqBBX13*, *CqBBX18*, *CqBBX19*, *CqBBX20*, *CqBBX21*, *CqBBX26*, *CqBBX28*, and *CqBBX31*, except for some genes down-regulated at a certain time point. Previous studies have shown that *A. thaliana* AtBBX1 protein regulated flowering pathways (Putterill et al., 1995), the apple MdBBX21 protein was associated with anthocyanin biosynthesis (Zhang et al., 2021), and some BBX proteins were associated with photomorphosis (Gangappa, Holm & Botto, 2013), while BBX genes have been less reported in plant drought resistance, such as Liu et al. (2019) have found that heterologous expression of *CmBBX22* in chrysanthemums can delay *A. thaliana* leaf aging and improve drought tolerance. We can speculate that BBX may have a large role in drought resistance in quinoa.

## Conclusions

In summary, the *CqBBX* genes of quinoa were analyzed at the genome-wide level, and a total of 31 *CqBBX* genes were identified. The analysis of the basic physicochemical properties, gene structure, conserved motifs, protein interaction, cis-acting elements, and expression patterns of *CqBBX* gene family members showed that *CqBBX* gene family members had conservative and diverse characteristics. In addition, the transcripts amounts of 13 *CqBBX* genes under three abiotic stresses of drought, salt, and low temperature were also studied. Among them, except for *CqBBX05*, the rest of the *CqBBX* genes can show different degrees of upregulation under PEG stress. This study provides a basis for further understanding of the role of BBX in quinoa growth and development and abiotic stress response.

##  Supplemental Information

10.7717/peerj.14463/supp-1 Table S1 The primer designed for qRT-PCR

10.7717/peerj.14463/supp-2 Table S2 Information of 88 BBX genes in this study

10.7717/peerj.14463/supp-3 Table S3 cis-acting elements in the promoter region of CqBBX genes

10.7717/peerj.14463/supp-4 Figure S1 Structural domains of CqBBX protein family members

10.7717/peerj.14463/supp-5 Table S4 The values of 31 CqBBX genes in 13 tissues downloaded from NCBI

10.7717/peerj.14463/supp-6 Table S5 The transcripts amounts of 13 genes by qRT-PCR

10.7717/peerj.14463/supp-7 Data S1 qRT-PCR raw data

## Additional Information and Declarations

### Data Availability

The following information was supplied regarding data availability: The raw measurements are available in the Supplementary Files.
