# Supplementary figures and images for: Genome-wide identification and expression pattern analysis of quinoa BBX family
Source: PeerJ. 2022 Dec 5;10:e14463. doi: 10.7717/peerj.14463 (PMC9745916; doi:10.7717/peerj.14463)

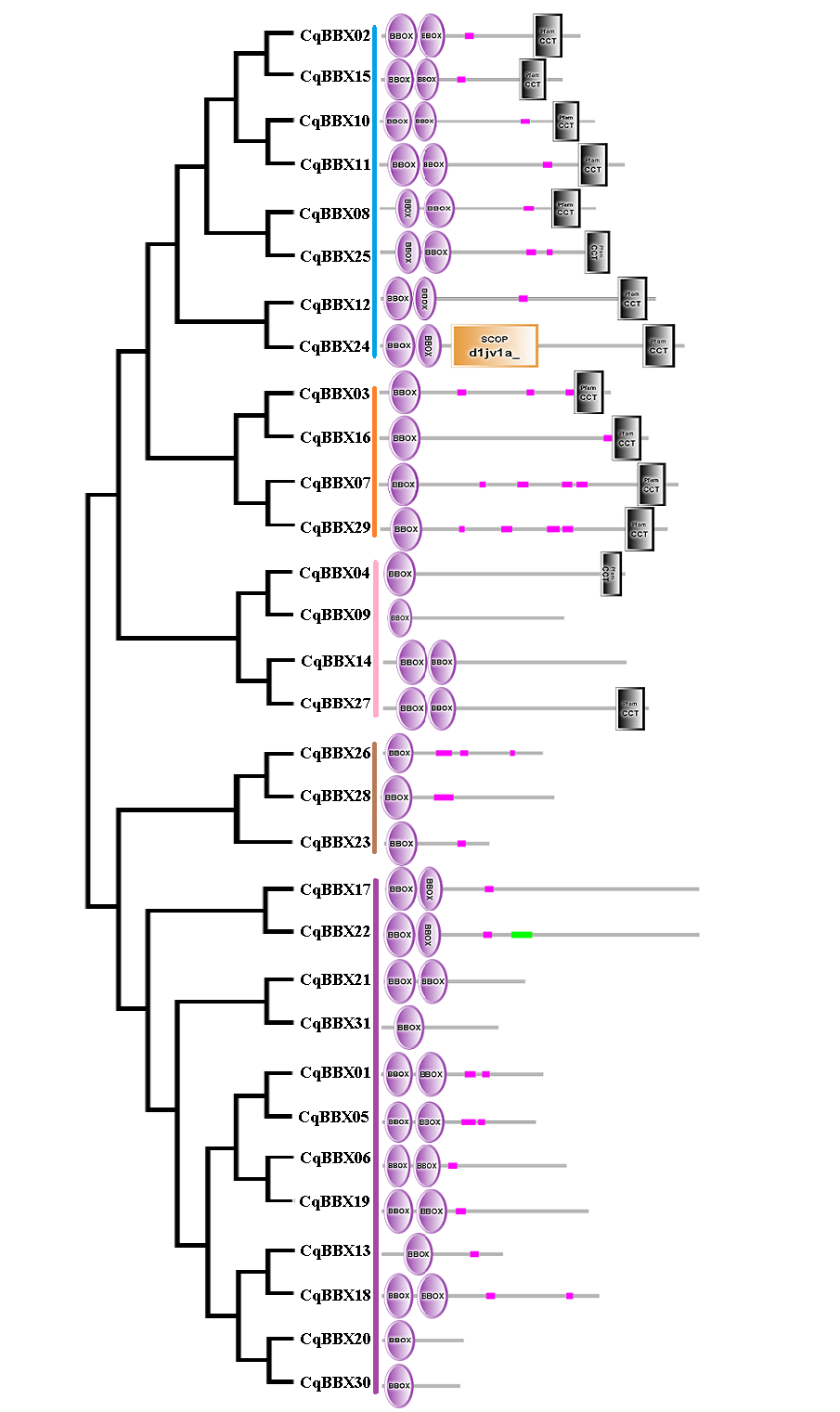

Supplement: Figure S1 [file peerj-10-14463-s004.png]
